# Trolleys, crashes, and perception—a survey on how current autonomous vehicles debates invoke problematic expectations

**DOI:** 10.1007/s43681-023-00284-7

**Published:** 2023-04-17

**Authors:** Suzanne Tolmeijer, Vicky Arpatzoglou, Luca Rossetto, Abraham Bernstein

**Affiliations:** 1 Information Systems, Socio-Technical Systems Design (WISTS), University of Hamburg, Vogt-Kölln-Straße 30, 22527 Hamburg, Germany; 2 Department of Informatics, University of Zurich, Binzmühlestrasse 14, 8050 Zurich, Switzerland

**Keywords:** Autonomous vehicles, Subjective ethics, Ethical dilemma, Ethics survey

## Abstract

Ongoing debates about ethical guidelines for autonomous vehicles mostly focus on variations of the ‘Trolley Problem’. Using variations of this ethical dilemma in preference surveys, possible implications for autonomous vehicles policy are discussed. In this work, we argue that the lack of realism in such scenarios leads to limited practical insights. We run an ethical preference survey for autonomous vehicles by including more realistic features, such as time pressure and a non-binary decision option. Our results indicate that such changes lead to different outcomes, calling into question how the current outcomes can be generalized. Additionally, we investigate the framing effects of the capabilities of autonomous vehicles and indicate that ongoing debates need to set realistic expectations on autonomous vehicle challenges. Based on our results, we call upon the field to re-frame the current debate towards more realistic discussions beyond the Trolley Problem and focus on which autonomous vehicle behavior is considered *not* to be acceptable, since a consensus on what the right solution is, is not reachable.

## Introduction

As autonomous cars start approaching our daily reality, there has been increased attention to how these cars should be programmed and what the consequences will be for drivers and other traffic participants. In academic research, different disciplines have focused on different aspects of the topic. While engineers are mostly focusing on the technical capabilities needed to increase autonomous vehicle (AV) autonomy (e.g., [1–3]), AI ethicists have started discussing moral dilemmas these cars might face and how they should be programmed to deal with them (e.g., [4–6]). Especially the use of human responses to ethical dilemmas is being explored as guidelines for AV programming [7–9]. The Moral Machine experiment [8] is the best-known example of this. On the experimental platform that went viral, participants from around the globe answered thirteen ethical dilemmas on autonomous cars. For each dilemma, there were two decision options: stay your course, saving passengers inside the car, or swerve and save pedestrians on the road. Different factors were varied in the experiment, including the age, fitness, and social status of the traffic participants in the dilemma. While the rich data set that resulted gave much insight into people’s ethical preferences for the tested dilemmas, the authors acknowledge some limitations, including that ‘*characters were recognized [...] with 100% certainty, and life-and-death outcomes were predicted with 100% certainty. These assumptions are technologically unrealistic, but they were necessary to keep the project tractable*’ [8, p 63].

In addition to the missing level of technical realism in these types of scenarios, there are additional issues with using ethical dilemma results as a starting point for AV regulations. Firstly, there is the question of the application value of lay people’s preferences. On the one hand, fitting the ‘participatory turn’ in the governance of science innovation [10], lay people should be involved in shaping AV guidelines to increase the chance of acceptance, as social norms influence acceptance of AVs [11]. On the other hand, interaction experience with AVs positively influences people’s perception of them [12], implying that their current attitudes will not reflect their preferences in the future and should, therefore, not be taken as a ground truth for AV policy. Meanwhile, the media spends much attention on negative aspects of AVs, such as AV crashes and unintended use of autopilots, which leads to more negative attitudes towards AVs [13]. As [14] argue, lay people’s AV preferences should only be considered in combination with expert insights, be screened for bias, and investigated for overall coherence, before any conclusions can be drawn for policy implications. While these surveys can give some insights into current sentiments, direct application beyond that should be called into question.

Furthermore, these highly simplified scenarios do not translate well to practice, where many more variables are combined during a time frame for decision-making (rather than one static point), at different levels of uncertainty and ambiguity, under time pressure, with more than just a binary decision option. Especially the use of discriminatory variables, such as gender and social status of a traffic participant [8] as well as life value hierarchy, are not only prohibited by law, but also unrealistic given current technical capabilities of AVs [15].

To highlight how quickly people’s responses can change based on the design of ethical dilemmas or framing of AV capabilities, we (i) expand the Moral Machine experiment to include a third decision option, time pressure, and a more realistic visual perspective of the presented scenarios, and (ii) present participants with different details and framing of AV performance, to see how this influences their perceptions.

Based on our findings, we argue that the focus on such simplified moral dilemmas in research and disproportionate framing of AV crashes in the media contribute to inaccurate expectations of both non-technical researchers and the general public on what an AV encounters on the road and what it can/should do, as they misapply though experiments and aim to turn them into general policy. We urge AI ethicists and engineers to collaborate more to see how these high level ethical insights could be used in practical coding of AVs and incorporated in specific framework, as well as argue for caution in the direct practical application of lay people’s current AV preferences.

In the remainder of this paper, we outline related work on AVs and ethics (Section 2). We present our research questions and hypotheses (Section 3) and describe the method (Section 4) and results (Section 5) for our experiment. We discuss the implications of our findings (Section 6), and conclude our work (Section 7).

## Related work

After a general overview of AVs and ethics, we discuss the Moral Machine experiment and other ethical dilemma surveys in more detail. We highlight the importance of limited time in AV decision-making, and how computers differ from humans in how they deal with this. Finally, we discuss the effect that media framing has on general perception.

### Autonomous vehicles and ethics

An autonomous vehicle, also known as an autonomous car or self-driving car [16–18], is a vehicle that can drive itself safely without assistance from a driver and with the ability to sense its surroundings [19].

There are six levels of driving automation as defined by the Society of Automotive Engineers, ranging from ‘No Driving Automation’ (level zero) to ‘Full Driving Automation’ (level five) [20]. The highest level available for purchase at the time of writing is level three: ‘Conditional Driving Automation.’ For example, Honda provides a Traffic Jam Pilot system which gives a car control over its brakes, steering, and throttle [21]. There are many challenges to the development of AVs with increased autonomy, including technical obstacles such as computational resources, non-technical issues like consumer trust, policy development, and social issues such as ethics for AVs [22]. To prepare for a future where AVs become part of everyday traffic and shape expectations and policy on time, there has been much discussion on the ethical aspects of AVs (e.g., [23–26]).

Most discussions on AV ethics rely on variations of the ‘Trolley Problem’ [27] — a series of thought experiments in which a human has to decide whether to, through inaction, allow a runaway trolley to kill five people on the track, or spare those people by actively swerving the trolley to a different track, killing one person instead. In the context of AVs, this has been framed in different contexts, such as deciding whether an AV should not hit a young girl on the road, but swerve at the expense of an elderly lady on the sidewalk [4]. The main argument for the relevance of these types of ethical considerations is that AVs will be able to process information more quickly and, hence, have to make rational predetermined decisions in situations where human drivers would have to act on split-second instincts [4]. However, technical challenges related to limited computational resources, efficient object detection, and an erratic environment [22] would indicate an AV may not be able to recognize traffic participant features on time for predetermined decision-making—at the very least, not in all possible cases. Nevertheless, there have been even more advanced discussions that not only assume that AVs will have ethical decision settings, accepting the mentioned premise that we can predetermine AV’s ethical dilemma guidelines, but also argue which setting the car should be in [5].

To raise public awareness of AV ethics and increase public acceptance through participation [8], one approach to finding appropriate ethical settings for AVs has been through the use of ethical preference surveys.

### AV ethics preference surveys

One of the most impactful works for ethical AV preferences thus far is the Moral Machine experiment [8]. The Moral Machine experiment utilized an online platform to gather millions of human decisions on moral dilemmas where an AV must choose between two action options — *swerve* or *continue*. During the experiment, participants had to judge which of two possible actions was considered more acceptable. The characters used in the scenarios had different features including their sex, age, social status (e.g., criminal, homeless, executive, athlete), fitness state, and whether they were human or animals. The authors found that the strongest global preferences, which held across different cultural groups, were to spare human lives over animals, spare more lives, and spare young lives.

Interesting results are also provided from other surveys. For instance, respondents’ moral preferences differ more under risky conditions than under uncertainty. Findings show participants prefer the AV to stay in their lane and do an emergency stop as a default action, independently of whether this produces maximum well-being in the situation at hand [9]. Additionally, more drivers preferred swerving under a level of uncertainty than under risk [9]. In yet another study, participants considered a more utilitarian response by an AV, i.e., choosing the option that saves more lives, to be the more morally acceptable choice [28].

The relevance of such ethical dilemma surveys is partially underlined by results of a related study, which showed that potential consumer adopters of AVs consider ethical dilemmas to be the most important and prominent issue to be addressed [29]. People accept and prefer other people to buy utilitarian AVs but in personal use they would like to use the ones that save their own lives [30]. This distinction in the expected behavior of AVs could even lead to a decrease in the overall acceptance of such vehicles [31].

### Thinking fast and slow

When considering such dilemmas and resulting policies for AVs, the reasoning is that dilemmas are considered beforehand or corrected afterwards by humans in a deliberate fashion, so that the AV can make fast decisions at the moment [32]. At this point, it is important to highlight that humans and machines do not reason in the same way. According to [33], humans have a distinction between intuitive, quick, and heuristic-oriented decision making (dubbed *‘system 1’*) and deliberative, logical, and rational decision making (*‘system 2’*). When they take longer, it can be an indication that the decision is harder to make [34]. Machines on the other hand do not have this distinction, or at least not in a clearly distinguishable way [32]. It is interesting to note that human instinctive action is accepted to have flaws stemming, e.g., from time-constrained reactions, whereas machines, even when they may be using a stateless or reactive model [35], are expected to follow an elaborate process of complete reflection. While this might sounds obvious, it impacts how humans would act themselves in crash situations and the expectations they have from machines such as AVs.

In general, people tend to judge humans more on their intentions and machines on the outcome [36]. Additionally, they tend to view machine actions to be more immoral and harmful when scenarios involve physical harm [36]. This is especially relevant in AV crash scenarios. Judges assign more liability to AVs and treat injuries caused by AVs more seriously than those caused by human drivers [37]. When participants were asked to judge crash decisions for AVs compared to human drivers, they preferred the AVs to minimize harm more than human drivers [38].

This calls into question whether human preferences on ethical surveys beforehand reflect their acceptance of actual AV decisions after a crash. In part, this is related to the fact that human drivers have to act based on *‘system 1’*, while AVs do not. To verify whether people’s ethical preferences really differ under time pressure, verification is needed of the influence of ‘thinking fast and slow’ on crash decisions.

### Issues with current AV ethics debates

Discussions on Trolley Problem variations for AVs give general insights into people’s initial response to AV dilemmas. However, there are various issues with this approach.

In earlier work, some of the Moral Machine experiment’s authors acknowledge the issues with Trolley Problems as being simplified scenarios compared to reality, and that real life provides statistical problems that should be solved instead [6]. Experts on both AI and ethics agree that while the Moral Machine experiment can serve as a starting point for discussions on AV ethics, there are many issues with it, including that participants’ own decision preferences were not studied and that trolley dilemmas are useful to pose questions but not to find answers regarding AV policy to be implemented. [39, 40]. [41] further argues that ethics for AVs as discussed in their current form are not relevant, among other reasons, because certainty and knowledge are assumed which is not likely in real life. He argues that rather than laying the focus on ‘what is considered right’, a question we will find no universal answer to, the focus should be on ‘what is not considered wrong’. Similarly, [42] argues that data collected by initiatives such as the Moral Machine experiment are not suitable as benchmarks for artificially intelligent agents, as such benchmarks incorrectly equate average participant preference with moral correctness.

Additionally, the framing of scenarios highly influences people’s responses. [38] found that framing scenarios from a pedestrian versus driver perspective changes people’s answers on the best possible AV actions. People are generally risk averse and weigh risks more heavily than benefits [29]. Inflated focus on car crashes in the news help emphasize the possible risks of AVs and influence AV perception [13, 43]. AVs are expected to decrease the number of car crashes tremendously, but such technological advances cannot come without any fatal crashes until AVs are fully deployed, something that according to some should not be underestimated [44]. Many such accidents thus far have happened due to the misunderstanding of the autonomy level of the driver [45]. This leads to the high importance of the driver being more informed and clearly understanding the AV system capabilities. Moreover, the few AV crashes have been overstated by the media, more than all other crashes and way beyond the positive progress in the performance of AVs [46]. [47] argues that the focus on behavior in crash situations can arguably also be detrimental to the overall safety of an AV, since it can pull attention away from what would be the correct behavior in mundane traffic situations. Everyday driving embodies trade-offs between values such as mobility, efficiency, and the consideration of pedestrians’ responsibility, all of which should be appropriately considered.

The current scientific debates and media coverage lead potential users to incorrectly assume (simplified) ethical dilemmas are the most prominent issue to solve for AVs [29]. Further confusion might stem from a misunderstanding on how the behavior of an AV comes to be. [48] described the training of AVs with the analogy of operand conditioning rather than a set of instructions in a classical algorithmic sense, and argues that it is therefore unclear how much weight should be put on trying to enforce a particular behavior in rare Trolley-Problem-like scenarios, given that this might even lead to other potentially negative consequences. If potential users have biased information about the functionality, operation, and behavior of AVs, they end up having an incorrect mental model of the system’s capabilities and challenges [49], which in turn affects people’s willingness to use AVs.

## Research questions

One major drawback of using variations of the Trolley Problem is that they are highly simplified. They include binary decision options, an observer perspective, and unlimited time to analyze the scenario. We vary different variables to test our hypotheses, which are described below.

### Dilemma perspective

**Fig. 1:**
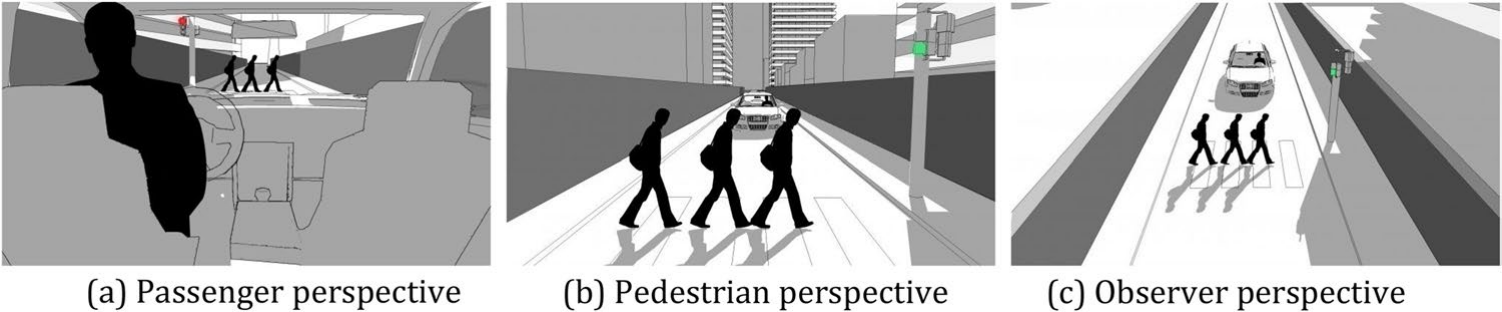
Example of presented dilemma scenario from three perspectives

Most moral dilemmas are presented through the comfort of the observer. Respondents are mainly watchers or witnesses of an accident that is about to happen. Of course, this situation creates comfort, but also a distance from the event. Observers have the opportunity to react or not to an event, which is otherwise quite impersonal to them. We query whether the preferences will stay the same, if it is the respondent’s life that is in danger. Moral preferences should be shaped in a way that reaches a consensus regardless of people’s perspectives on accidents. This leads us to our first research question:

- **RQ1. Does the perspective of the dilemma (see Fig. ****1****) change participants’ preferences?** We expect that (H1) a pedestrian perspective will lead to more swerve preferences than a driver perspective, and (H2) that an observer perspective will lead to more abstaining from a decision.

### Time pressure

In case of an impending accident, a driver (be it human or AV) needs to decide how to act in a very short amount of time. A starting point for human acceptance of AV decision is to compare AV actions with how human drivers would act in the situation themselves — a component that the Moral Machine experiment was criticized for not including [39]. Additionally, it is possible that *‘system 1’* and *‘system 2’* are utilized, depending on time pressure for a decision [33]. As time pressure decreases consistency in the decision-making patterns and an algorithmic decision is preferable to a human decision under high time pressure [50], we evaluate how time pressure affects moral preferences and what variations are observed between moral preferences under time and no time pressure. To this end, we formulate the following research question:

- **RQ2. Does ‘thinking fast and slow’, i.e., time pressure, change participants’ preferences?** We expect that (H3) participants are more likely to swerve under time pressure.

### Non-binary decision options

Many ethical preference surveys give a binary decision option: the participant can swerve or continue. While this forces participants to pick a preference, this also gives biased results: for some dilemmas, they might have a clear preference, while for others, they actually do not but are forced to pick. For this reason, we add a third option: *‘no preference’*. This allows us to verify whether found results in other ethical surveys are really such strong preferences as presented.

### More realistic depiction

We deploy a more realistic drawing style to depict scenarios. Different surveys, such as [8, 38], employ an almost cartoon-like style in their scenarios. Possibly, by employing a more realistic style, the scenarios become less abstract, leading to different participant preferences. Furthermore, we explicitly construct scenarios where an emergency stop cannot be used. This is needed since some results indicate that people would prefer an emergency stop, independent of the scenario [9]. This leads to our third research question:

- **RQ3. Does a slightly more realistic dilemma presentation lead to different results compared to other ethical preference surveys (e.g., **[8, 9, 28]**)?** We expect that (H4) a non-trivial amount of people will abstain from a decision when they have the option, but (H5) still have a preference for saving lawful traffic participants and saving more lives.

### Capabilities framing

In addition to our approach to ethical preference surveys, we want to investigate the effect that framing has on participants’ AV impressions. We hypothesize that because potential users do not have clear expectations yet of what AVs can do and the exact benefits it would bring, they are highly influenced by the framing of AV information. This is discussed in our final research question:

- **RQ4. Does framing of AV capabilities change participants’ preferences?** We expect that (H6) participants are less likely to want to use an AV when crash statistics are emphasized (i.e., using negative framing), but that (H7) participants are more likely to want to use it when those statistics are placed into perspective by human crash statistics (i.e., positive framing).

## Method

In the following section, we introduce our experimental design, and its implementation. Our experiment follows a 3×2 between-subjects design: participants got assigned one of three possible perspectives (driver, pedestrian, or observer) and one of two possible time options (unlimited or time-restrained).

To set realistic time pressure where participants could still process all details of the questions, we pretested how long participants would take for the various scenarios. In this pretest (*N*=15), the average time for all respondents in all perspectives was 15.5 seconds. Hence, we forced time-limited responses to a maximum of 15 seconds, to create a slight time pressure but also ensure that the majority of people can answer within this time.

Data was gathered using a survey, carried out via Qualtrics.^1^ In the first part of the survey, demographic questions were asked, as well as their affinity for technology (ATI) using the validated ATI scale [51]. In the next part, respondents had to give their preferences for eight ethical dilemmas, according to their assigned perspective and time pressure. To research RQ1, participants are assigned one of three different dilemma perspectives, namely: passenger, pedestrian, or observer. The different perspectives can be found in Fig. 1. Compared to [38], we employ a less immersive but more realistic style, to verify whether dilemma perspectives indeed make a difference in decision preferences. For each perspective, images differed according to the a) the number of people (one vs. more; depicted as one vs. three people) and b) the color of the traffic light (red vs. green). The scenarios were presented in a randomized order. In the third part of the survey, participants were asked to indicate how likely it is for them to use the described AV in six different scenarios on a 5-point Likert scale. The information for each question was framed in such a way that information focused firstly on neutral components (i.e., two scenarios with information on technical capabilities), then negative components (i.e., two scenarios with crash information), and finally, positive components (i.e., two scenarios with benefits of AVs and crash statistics compared to human drivers). The order of these six scenarios was fixed, to be able to investigate the order effects as mentioned in H6 and H7. Lastly, to get more information about participants experiences and preferences, follow-up questions were presented regarding the Oxford Utilitarian Scale [52], driver license, frequency of driving, car ownership, automation level of participant’s car as well as vehicle crash history.

To promote transparent and open science practices, we make the full survey contents as well as anonymized gathered data available via the Open Science Foundation.^2^

Participants were recruited via crowd-sourcing platform Prolific.^3^ This happened three times: for the mentioned time-pressure pretest (*N*=15), a general pretest to test our design (*N*=30), and the final experiment. For all instances, participants were paid according to Prolific’s suggested hourly rate of GBP 7.52. To ensure quality of work, the following filters were applied: participants had to be fluent in English and have an approval rate of at least 85% for at least 10 completed tasks. A power analysis based on the pretest results (expected effect size 0.25, *α* error 0.05, power/1-*β* error 0.95, numerator degrees of freedom 10, number of groups 6) resulted in 400 participants needed for the experiment [53].

## Results

We conducted the survey in November of 2021 and received a total of 406 valid responses. Out of the surveyed participants, 199 self-identified as female, 203 as male, and 4 self-identified otherwise. The age of the participants ranged from 18 to 81 years, with a median of 29 and an average of roughly 32.3 years. The participants were each assigned roughly equally to one of the previously described perspectives; the exact distribution is shown in Table 1.

**Table 1:** Group sizes per perspective and time pressure

	Time pressure		
	With	Without	Σ
Passenger	67	70	137
Pedestrian	63	70	133
Observer	68	68	136
Σ	198	208	406

### Preferred actions

Across the 8 scenarios presented to each participant, they collectively expressed their preference (or lack thereof) for the action which the car is to take in 3158 instances. Scenarios where participants placed under time pressure did not answer in time were not counted. Across all evaluated scenario instances, the participants expressed a preference for the car to swerve, thereby sparing the pedestrians and risking the well-being of its passengers in 62.5% of instances. In 18.6% of cases, participants preferred for the car to continue in its lane and in the remaining 18.9% of cases, the participants did not express a preference for an action. We therefore already see support for H4, as participants used the option to not express an explicit preference in almost one out of every five opportunities. Table 2 shows the breakdown of these preferences with respect to perspective and time pressure. The ratios of expressed preferences are very similar across the different perspectives and we did not find a statistically significant difference between them.

**Table 2:** Preferred actions per perspective, showing the sum of expressed preferences both with and without added time pressure

	Preferred action		
	Swerve	Continue	No preference
Passenger	303 + 369 63.3%	95 + 115 19.8%	103 + 76 16.9%
Pedestrian	312 + 345 63.5%	78 + 108 18%	84 + 107 18.5%
Observer	347 + 298 60.7%	80 + 111 18%	92 + 135 21.3%
Σ	962 + 1012 62.5%	253 + 334 18.6%	279 + 318 18.9%

Table 3 shows the distribution of preferred actions with respect to the color of the traffic light from the perspective of the car as well as the ratio between pedestrians crossing the road and passengers in the car. We can see that, while swerving is the dominant action preference in all cases, the ratio

**Table 3:** Preferred actions per color of the traffic light from the car’s perspective and ratio of pedestrians on the street versus passengers in the car

		Preferred action		
		Swerve	Continue	No preference
More	Red	215 (81%)	11 (4%)	40 (15%)
	Green	345 (66%)	93 (18%)	85 (16%)
Equal	Red	532 (68%)	91 (12%)	159 (20%)
	Green	450 (57%)	180 (23%)	165 (20%)
Fewer	Red	308 (59%)	119 (23%)	93 (18%)
	Green	124 (46%)	93 (34%)	55 (20%)
Σ	Red	1055 (67%)	221 (14%)	292 (19%)
	Green	919 (58%)	366 (23%)	305 (19%)

Towards swerving increases when there are more pedestrians than passengers in a scenario or when the car has a red light. This indicates that the survey participants have a preference towards lawful behavior (see also ‘Green’ in Tables 4 and 5) as well as a preference towards minimizing the number of people involved in a collision (see also ‘Fewer’ and ‘More’ in Tables 4 and 5), thereby behaving in accordance with H5. This is also supported by the fraction of participants having no preference for an action being highest in the scenarios where the number of passengers and pedestrians are equal.

**Table 4:** Coefficients, value ranges and *z*-test values for logistic regression model predicting the probability of survey participants preferring the *swerving* action for any given scenario

Parameter	Coef	Range	Std. err	*P*(> z)
Intercept	0.522	**0.522**	0.295	0.077
*Age*	0.008	**0***.***144** to **0***.***648**	0.002	**0***.***0001**
*Male*	− 0.016	− 0*.*016*,*0	0.231	0.94
*Female*	− 0.001	0*,*0*.*001	0.233	0.999
*ATI*	− 0.023	− **0***.***138** to − **0***.***031**	0.027	0.387
*Utilitarian*	− 0.007	− **0***.***441** to − **0***.***07**	0.003	**0.009**
*Fewer*	− 0.259	− **0***.***259***,***0**	0.056	** < 10**^**– 5**^
*More*	0.303	**0***,***0***.***303**	0.058	** < 10**^**– 6**^
*Green*	− 0.348	− **0***.***348***,***0**	0.048	** < 10**^**– 12**^
*Passenger*	0.069	**0***,***0***.***069**	0.056	0.219
*Pedestrian*	0.072	**0***,***0***.***072**	0.057	0.204
*Time*	0.092	**0***,***0***.***092**	0.046	**0.047**

**Table 5:** Coefficients, value ranges and z-test values for logistic regression model predicting the probability of survey participants preferring the *continue* action for any given scenario

Parameter	Coef	Range	Std. err	P(> z)
(Intercept)	− 0.566	− **0.566**	0.333	0.089
*Age*	− 0.013	− **1***.***053** to − **0***.***234**	0.003	< **10**^**– 6**^
*Male*	− 0.042	− **0***.***0420**	0.255	0.868
*Female*	− 0.193	− **0***.***193***–***0**	0.257	0.453
*ATI*	− 0.023	**− 0***.***138** to − **0***.***031**	0.031	0.466
*Utilitarian*	− 0.0007	− 0*.*0441 to − 0*.*007	0.003	0.825
*Fewer*	0.42	**0, 0.42**	0.063	** < 10**^**– 10**^
*More*	− 0.256	− **0.256, 0**	0.07	**0.0002**
*Green*	0.47	**0, 0.47**	0.056	** < 10**^**– 16**^
*Passenger*	0.067	0, 0.067	0.065	0.304
*Pedestrian*	0.042	0, 0.042	0.066	0.531
*Time*	− 0.098	− **0.098, 0**	0.054	0.069

### Combined factors

To determine the relative influence of the different factors on the preferred action in each scenario, we perform a logistic regression analysis to predict the probability of a participant actively choosing to swerve or to continue over expressing no explicit preference. We use the following parameters as input to the model. Since not all parameters have the same value range, we report the minimum and maximum value for each parameter as well.

*Age:* the age of the participant in years, ranging from 18 to 81. *Male:* 1 if the participant identifies as male, 0 otherwise.

*Female:* 1 if the participant identifies as female, 0 otherwise.

*ATI:* affinity for technology [51] ranging from 1.33 to 6.

*Utilitarian:* Oxford Utilitarian Scale [52] raging from 10 to 63.

*Fewer:* 1 if there are fewer pedestrians on the road than passengers in the car, 0 otherwise.

*More:* 1 if there are more pedestrians on the road than passengers in the car, 0 otherwise.

*Green:* 1 if the traffic light for the car is green and the car has the right of way, 0 otherwise.

*Passenger:* 1 if the scenario is presented from the passenger’s perspective, 0 otherwise.

*Pedestrian:* 1 if the scenario is presented from the pedestrian’s perspective, 0 otherwise.

*Time:* 1 if the participant was put under time pressure when answering, 0 otherwise.

Tables 4 and 5 show the parameters and accompanying value ranges as well as their standard errors and *z*-test values for the preference to swerve or continue, respectively. Z-test values below 0.05 and value ranges with a magnitude of above 0.05 are highlighted in bold. We consider parameters fulfilling both criteria as relevant. For categorical parameters that represent multiple distinct possible options (such as red/green for the stoplight or passenger/pedestrian/observer for the scenario perspective), a one-hot encoding is used. This type of modeling then only uses N-1 options as an input to forego redundant information. This implies that lawfulness is represented by ‘Green’ and possible perspectives by ‘Passenger’ and ‘Pedestrian’.

When looking at the model to predict swerving, the parameters that fulfill both criteria are the participant’s age and utilitarian score, the ratio of pedestrians and passengers, the color of the traffic light, as well as the applied time pressure. Comparing these parameters between the two models, we can see that the sign of their respective coefficient flips for all but for the utilitarian score, which fails to fulfill either of the selection criteria in the model predicting the *continue* action. Age, traffic light color, and the ratio between passengers and pedestrians remain relevant, whereas the time component slightly exceeds the threshold. Since the perspective does not appear to have a relevant effect on the participant’s preference, we can reject H1 (see ‘Passenger’ and ‘Pedestrian’ in Tables 4 and 5). H3, however, is supported by the observations, since the presence of time pressure increases the probability of participants choosing to swerve (see ‘Time’ in Table 4).

When performing an equivalent analysis trying to predict instances where a participant did not express a preference, we do not find any scenario-specific parameters with positive and significant coefficients. We omit the table of these non-significant results for the sake of brevity. Based on this lack of a finding, we can reject H2. Despite the fraction of responses with no expressed preference being highest for the observer perspective as shown in Table 2, the perspective does not appear to be a significant influencing factor when considering the other parameters.

### Framing effects

In addition to the crash scenarios described above, the third part of the survey also presented six descriptions of scenarios involving autonomous cars and ask participants to describe how likely they would rate it for them to use the described vehicle on a 5-point Likert scale. Out of these 6 scenarios, two are worded neutrally, two have a positive framing, and two have a negative framing. All presented situations are based on current real-world data. Given the non-normal distribution, we use a Wilcoxon signed-ranks test to see which responses can be considered different. Because of our designed order effect, we compare scenario scores with scores of the previous scenario to investigate what effect the added information and framing had on perception. In Table 6, we show the normalized mean response per scenario and framing as well as the results of the statistical comparison. Especially the Wilcoxon results between framing blocks are relevant, i.e., between scenarios 2 and 3, and between scenarios 4 and 5 (displayed in bold). We find that, despite the scenarios all referring to real-world situations in which the vehicles demonstrate comparable performance, the positive or negative framing has a noticeable effect on the mean response of the surveyed participants. This thereby supports both H6 and H7.

**Table 6:** Mean reported likelihood of survey participants using an autonomous car with respect to the framing of the scenario

Scenario	Framing	Response				Wilcoxon		
		Mean		Median		*T*	*Z*	*p*
1	Neutral	0.489	0.556	0.5	0.75	1510	− 9.275	< 0*.*001
2		0.618		0.75				
						**3372**	− **11.369**	< **0***.***001**
3	Negative	0.378	0.427	0.25	0.5			
						7268	− 5.954	< 0*.*001
4		0.475		0.5				
						**3080**	− **11.193**	< **0***.***001**
5	Positive	0.680	0.686	0.75	0.75			
						4721	− 1.388	0.132
6		0.692		0.75				

Wilcoxon signed-rank test results are compared to the answer distribution of the previous scenario

## Discussion

In this section, we discuss the implications of the results found.

### Influence of dilemma perspective

Earlier work [38] showed that when ethical dilemmas for AVs were presented from the pedestrian perspective, participants were more likely to suggest selfpreserving actions. However, we find no difference in action preferences based on the presented perspective. One possible explanation can be that [38] used a virtual reality environment while we used static images to depict the scenarios.

This comparative finding has quite some serious consequences for the value of most AV ethics surveys. After all, if people’s reported preferences in such surveys, which mostly employ pictures to describe scenarios, do not reflect people’s preferences in more realistic settings such as VR, we can only assign limited value to them. The current usage of ethical preference surveys can still have value, in the sense that it can raise awareness with the general public, but its results cannot be trusted at face value.

### Framing of AV capabilities

Both our hypotheses regarding AV framing were supported by our experiments: people are less likely to want to use an AV when crash statistics are shown, while they become more likely to use them when those statistics are put into perspective. Except for the information shown in the last question, the answers had a different distribution for each new question. In other words, the information and framing of the questions highly influenced people’s perceptions and preferences. This strong framing effect also emphasized that lay people’s reported acceptance of AVs and intention to use is not stable, but depends on how the AV and its capabilities are framed.

This seems to be a direct consequence of the behavior-intention gap [54], where people often report different predicted behavior than they actually show in the same situation. Additionally, current technology development, like with AVs, is happening *“without a sound cultural framework that could give technology a sense beyond mere utilitarian considerations.”* [55, p 1] A fear response to the unknown is a way to make sense of incomplete information being presented—the general public would benefit from more realistic and complete information provision regarding AV capabilities.

### Time pressure makes a difference

The found results confirm our hypothesis that participants are more likely to swerve under time pressure. The effect of *‘system 1’* and *‘system 2’* are visible in our results, where the instinctive response to an object on the road is to swerve. This also confirms results by [56], where less available time led to more avoidance behavior, displayed by swerving actions.

This distinction between ‘thinking fast and slow’ can have consequences for the judgment of AV actions. The described related work showed that AVs are judged in a more utilitarian way and people expect AVs to undergo a complete, ‘rational’, and predetermined decision process. However, human drivers are excused for split-second decisions. Their heuristic response is to swerve, while they prefer the heuristic of the AV to be an emergency break, independent of the situation [9]. Related to the results of RQ4, framing the capabilities of the AV in a realistic manner—including what it can and cannot do under time pressure—could alter the judgment people have for AV decisions. It is therefore important that—in so far as preference surveys are used as input for the behavior of AV’s at all—survey participants are being educated about the commonalities and, more importantly, the differences between human and AV accident scenarios. 

### Influence of non-binary decision options

Compared to the Moral Machine experiment, we find similar results in terms of saving more lives and saving lives of traffic participants that adhere to the law, thereby supporting H5.^4^ However, our results also show that for nearly one in five scenarios, users choose the ‘no preference’ option, rather than swerve or continue, supporting H6. Despite there clearly being situations where the surveyed participants did not choose to express a preference, we were not able to identify any scenario-specific properties that would lead to such a lack of preference. Based on the collected data, we cannot say if there are factors that would lead participants to consistently not have a preference in certain scenarios or if the decision-making process of the participants has an inherently random component. As such, we have to reject H2 based on our data. Deeper insights are needed here, to see if certain personal traits of the participants can explain people answering ‘no preference’ over making a decision.

Nevertheless, just adding one more decision option already changed responses considerably, and gave more insights and details compared to existing results. Again, this calls into question what value we can give to decision scenarios with only two possible options, since i) more than two options are possible in most real-world scenarios, and ii) only providing two answers, therefore forcing people to choose, results in partially biased results. Since the Moral Machine experiment and similarly structured surveys explicitly force people to make a binary value judgment, even in cases where no such judgment needs to be made or must not be made [57], it is not a suitable tool for determining actionable behavior preferences.

### Stay away from the trolley

The various results discussed in this section show that the current approach to ethics for AVs is somewhat problematic. Specifically, the usage of intricate trolley dilemmas with minute traffic participant characteristics do not and should not directly influence AV policies. Moreover, since the framing of scenarios and AV capabilities have such a large impact on potential user perception, information for the general public, as well as academic discourse, should be designed to represent realistic assumptions about AV capabilities and challenges.

Based on our results, we specifically argue that future discussions on AV ethics and capabilities should take the following into account:

- Simplified Trolley Problems with binary options give unrealistic expectations of AV challenges.
- Results of ethical preference surveys for AV should be approached with caution when discussing possible AV policies.
- People are highly sensitive to the framing of AV capabilities and ethical dilemmas options. As such, any discussions on AVs should be informed by the current technical state of the art and the challenges that come with it.

The few generalizable results that were found across different surveys in different settings are that people prefer to save more people and save lawfully behaving people. This can definitely be a starting point for discussions regarding AV policies. However, for further discussions and insights on AV policies, the discussion needs to move towards a more realistic framing of the current challenges. We believe this can be achieved in different ways:

- When lay people are asked for their opinion, scenarios should be closer to realistic settings. This can be achieved by adding more decision options, time pressure, and an interactive 3D environment (such as TrolleyMod [58]).
- When interpreting the results, they should be combined with expert insights and participant traits. Given the observed influence on people’s decisions by the additions of simple variables such as a neutral option or time-pressure, it would be irresponsible to draw conclusions from these preferences directly.
- Any framing of AV challenges and questions, be it in research or media context, should be realistic and transparent regarding the capabilities of AVs and broader than simple trolley-problem-like scenarios. As argued by [59], there are also substantial differences between such dilemma scenarios and real-world traffic situations which make them ethically dissimilar. This dissimilarity needs to be carefully considered when drawing any conclusions from related surveys.
- Following [41], society is unlikely to ever agree on a universal set of ethics guidelines that fit everyone’s preferences. Instead, the debate should focus on what we consider unacceptable (and potentially unlawful in the future).

## Conclusion

In this work, we analyze the current debates on ethical decision-making for autonomous vehicles. Specifically, we argue that the focus on variations of the Trolley Problem in ethical preference surveys is problematic, because it gives unrealistic expectations of AV capabilities and challenges and this theoretical approach gives limited empirical insights. To this end, we run an ethical preference survey where we include more realistic features, such as different perspectives of the scenario, time pressure, and non-binary decision options. Additionally, we offer different framings of AV capabilities, to investigate how they influence user acceptance. We find that we do not replicate earlier findings that the dilemma perspective has an effect, but report that time pressure and non-binary decision options influence results compared to current ethics surveys. Furthermore, the framing of AV capabilities has a direct influence on user preferences. Our results underline the care we need to take when interpreting ethical survey results and that such surveys are not a suitable tool for directly determining AV policy. We call upon the field to re-frame current discussions to focus on realistic settings and challenges, to both have more practical insights into AV decision-making and set realistic expectations on AV capabilities and to not rely on preferences expressed in abstract and theoretical scenarios without first developing appropriate frameworks to think about how to incorporate such preferences into any conclusions related to tangible policy.

## Data Availability

Data and code generated during the analysis for this paper are available via https://osf.io/3cqrx.

## Footnotes

1 https://www.qualtrics.com.

2 https://osf.io/3cqrx.

3 https://prolific.co.

4 Given that we do not include discriminatory features in the scenarios, we cannot compare age, ‘social status’, or similar preferences.
